# From the Skin to Small Bowel: Metastatic Melanoma as a Hidden Source of Gastrointestinal Bleeding

**DOI:** 10.7759/cureus.94048

**Published:** 2025-10-07

**Authors:** Leonid Drober, Yosor Fiesal, Sa'd Sayida

**Affiliations:** 1 General Surgery, Rambam Medical Center, Haifa, ISR

**Keywords:** gastrointestinal bleeding, laparoscopic resection, metastasis, ­skin cancer, small bowel melanoma

## Abstract

Malignant melanoma involving the small bowel, whether primary or metastatic, is rare and often remains undiagnosed during life due to its asymptomatic course or nonspecific clinical presentation. Here, we report the case of an 81-year-old man with a history of spindle cell melanoma of the scalp, treated five years earlier, who presented to the emergency department with loss of consciousness. Laboratory tests revealed severe anemia (hemoglobin: 4.2 g/dL), and rectal examination demonstrated melena. Upper and lower endoscopy failed to identify a bleeding source. Abdominal CT showed a circular filling defect in the proximal jejunum. Subsequent enteroscopy revealed an ulcerated, friable mass occupying more than 50% of the luminal circumference. The patient underwent laparoscopic segmental resection of a 10 cm jejunal segment containing the lesion, followed by primary anastomosis. Histopathological analysis confirmed metastatic malignant melanoma infiltrating the intestinal wall. Small bowel melanoma is a rare but important cause of gastrointestinal bleeding. In patients with a history of melanoma presenting with unexplained anemia or overt bleeding, small bowel metastasis should be considered. Surgical resection offers both diagnostic confirmation and effective treatment.

## Introduction

Malignant melanoma is one of the most common solid tumors to metastasize to the gastrointestinal (GI) tract, with cutaneous lesions being the most frequent source of GI metastases. Metastatic involvement most commonly affects the small bowel, followed by the colon, stomach, and anorectum. The median interval from primary cutaneous melanoma diagnosis to GI metastasis is typically several years, with reported ranges from months to decades, underscoring the need for long-term vigilance in melanoma survivors [1,2].

Primary melanoma of the GI tract is rare, with the majority of GI melanomas representing metastases rather than true primary mucosal tumors. When primary GI melanoma does occur, it is most often found in the anorectal region, and less commonly in the small intestine or colon. These primary lesions are frequently asymptomatic in early stages and are often diagnosed only when complications such as GI bleeding, obstruction, or perforation arise [3,4].

Compared with cutaneous melanoma, primary intestinal melanoma generally presents at a more advanced stage and is associated with a higher rate of lymph node and distant metastases at diagnosis [5]. Its metastatic spread is characterized by early and aggressive regional lymph node involvement, often accompanied by simultaneous distant dissemination. In contrast, cutaneous melanoma more commonly demonstrates a stepwise progression from regional nodes to distant sites [6].

Dissemination of melanoma to the GI tract typically occurs via hematogenous spread from a primary cutaneous lesion [7]. The timing of GI metastases is highly variable, with a median interval of 34-65 months after initial melanoma diagnosis [8].

Symptoms are often nonspecific and may be subtle or absent until complications arise. Common symptoms of GI metastases include abdominal pain, dysphagia, small bowel obstruction, hematemesis, and melena. Abdominal pain and obstructive symptoms (such as nausea, vomiting, and intussusception) are frequent, especially with small bowel involvement. GI bleeding may present as hematemesis or melena, and is often the most common presenting symptom, either as frank or occult bleeding leading to anemia [3].

In patients aged 59-70 years presenting with abdominal symptoms, small bowel melanoma should be included in the differential diagnosis. Furthermore, in individuals with a prior history of malignant melanoma, the onset of altered bowel habits, intestinal obstruction, or GI bleeding should raise suspicion for small bowel metastasis.

The recommended preoperative assessment for detecting small intestine metastases in a patient with a history of surgically treated malignant melanoma should begin with a thorough clinical evaluation and laboratory assessment for anemia and coagulopathy. Imaging strategy should prioritize positron emission tomography/computed tomography (PET/CT), as it is superior to CT alone for detecting small bowel melanoma metastases. Video capsule endoscopy and enteroscopy can be considered for direct visualization and biopsy, particularly when imaging is inconclusive or for evaluation of occult bleeding [9].

Curative resection of small bowel metastases is indicated in patients with limited disease, good performance status, and absence of widespread extra-abdominal metastases. Surgery also provides effective symptom control for bleeding and obstruction, and may facilitate subsequent systemic therapy with immune checkpoint inhibitors, which further improves outcomes [10].

Our case highlights a patient with a history of surgically treated malignant melanoma of the scalp who presented several years later with overt GI bleeding, ultimately found to be due to a metastatic lesion in the small intestine.

## Case presentation

An 81-year-old man diagnosed with a grade III spindle cell melanoma of the scalp a few years ago underwent resection and sentinel lymph node biopsy (SLNB) of a superficial spreading melanoma on his scalp. He received adjuvant radiation and biological treatment with pembrolizumab from 2015 until discontinuation in 2017. The patient remained under oncological follow-up and underwent a PET/CT scan in 2018, which indicated no evidence of active disease.

Five years later, he was referred to the emergency room (ER) due to a head injury as a result of loss of consciousness and a fall to the floor. Lately, the patient had experienced several episodes of loss of consciousness; however, upon further questioning, he denied any evidence of rectal hemorrhage or reports of melena. This suggested the possibility of occult bleeding, which may have gone unnoticed until it progressed to severe hemorrhage, ultimately leading to syncope. On examination, he was pale but hemodynamically stable. After a head CT ruled out traumatic injury, the blood work showed anemia with a hemoglobin level of 4.2 g/dL. As part of the initial investigation, a rectal examination was performed, which revealed melena. A nasogastric tube was then inserted, but there was no evidence of coffee ground or fresh blood. The patient received two units of blood and was admitted to the surgical department for further evaluation.

**Figure 1:**
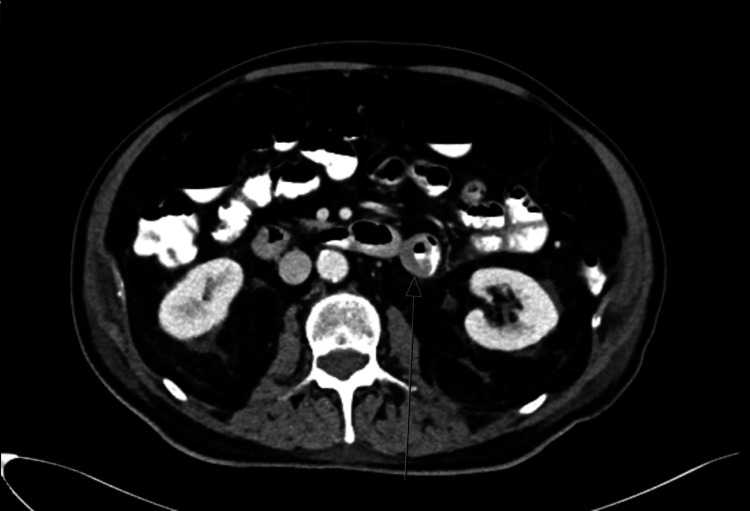
CT scan demonstrating a circular filling defect in the proximal jejunum, raising suspicion that this may be the source of the gastrointestinal bleeding.

**Figure 2:**
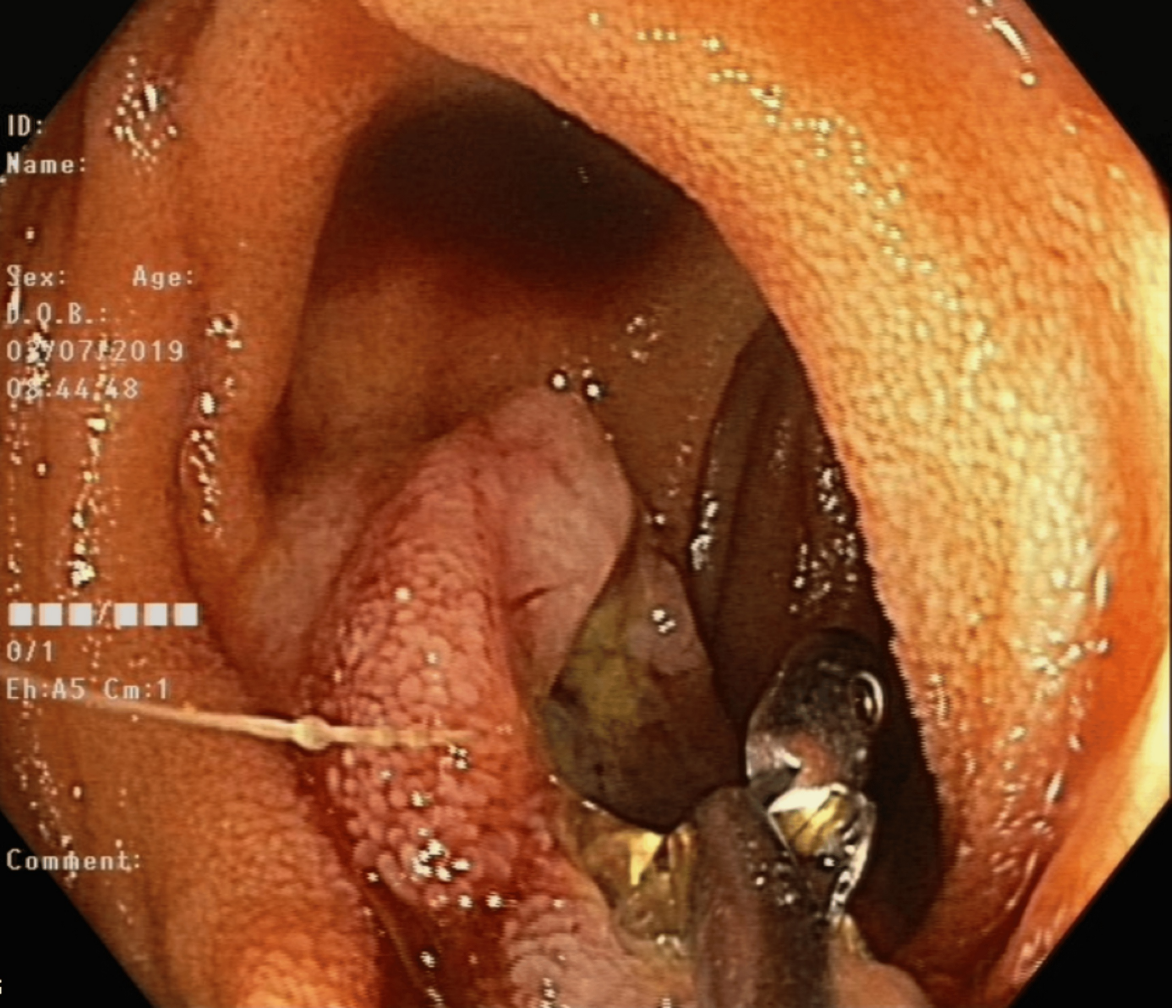
Enteroscopy showing an ulcerated, friable mass in the proximal jejunum located in the same region identified on the previously mentioned CT scan.

During the admission, the patient underwent a comprehensive workup, beginning with endoscopic investigations, including gastroscopy and colonoscopy, which did not reveal any source of bleeding. However, given the previously mentioned details regarding active bleeding and findings from the rectal examination, along with a noted decrease in hemoglobin levels, the patient subsequently underwent an abdominal CT scan to identify the source of bleeding. The abdominal CT scan showed a circular filling defect in the proximal jejunum of approximately 2.5 cm in diameter. Air in the center of the lesion was indicative of an ulcerative lesion (Figure 1). Consequently, we deemed it necessary to conduct an endoscopic examination through enteroscopy to obtain a definitive diagnosis before contemplating surgical intervention. An enteroscopy revealed an ulcerated, friable mass that occupied more than 50% of the luminal circumference in the proximal jejunum (Figure 2). It was biopsied and marked with a tattoo. The biopsy specimen revealed metastatic malignant melanoma.

**Figure 3:**
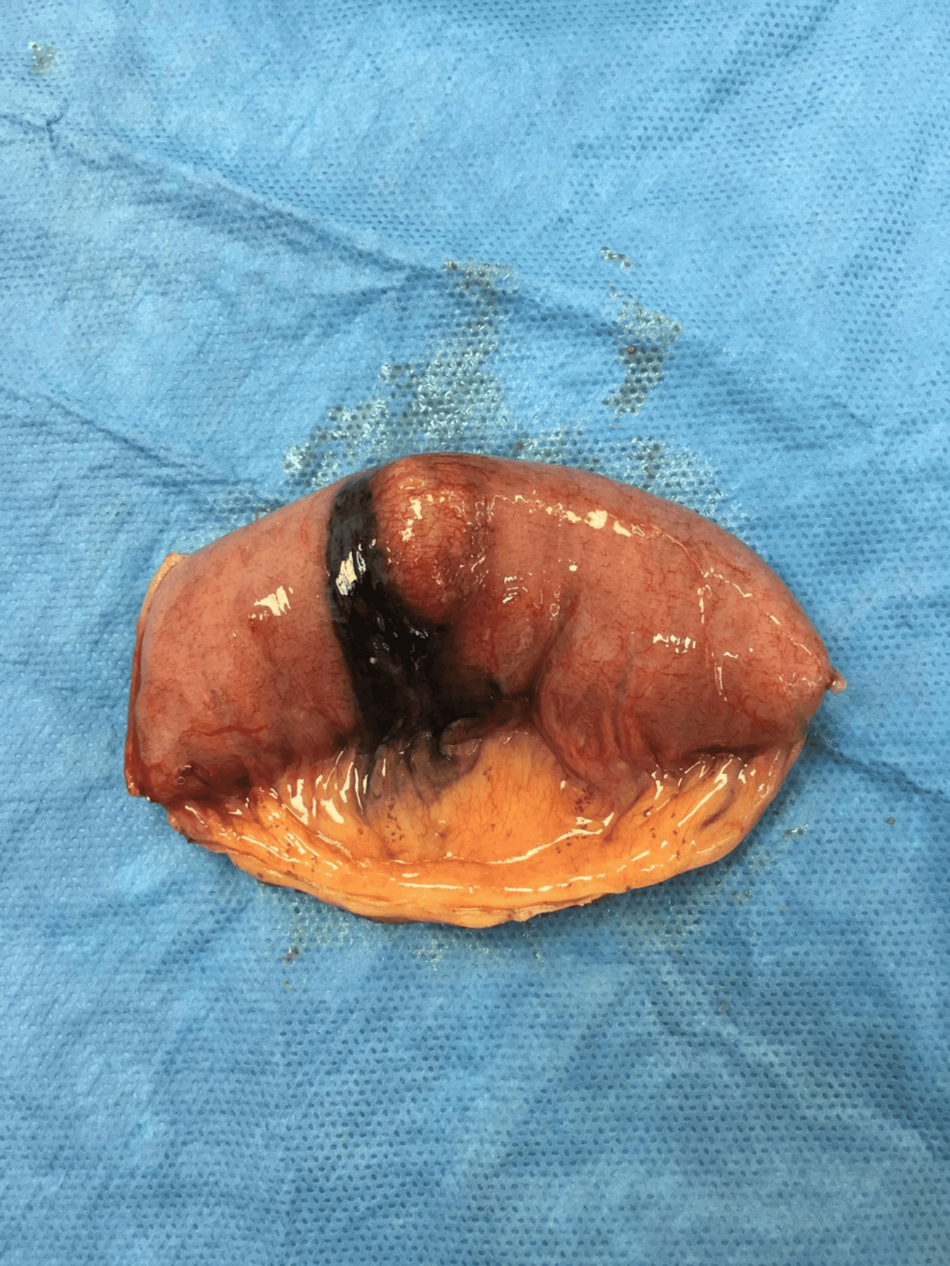
Segmental resection of the jejunum containing the lesion.

**Figure 4:**
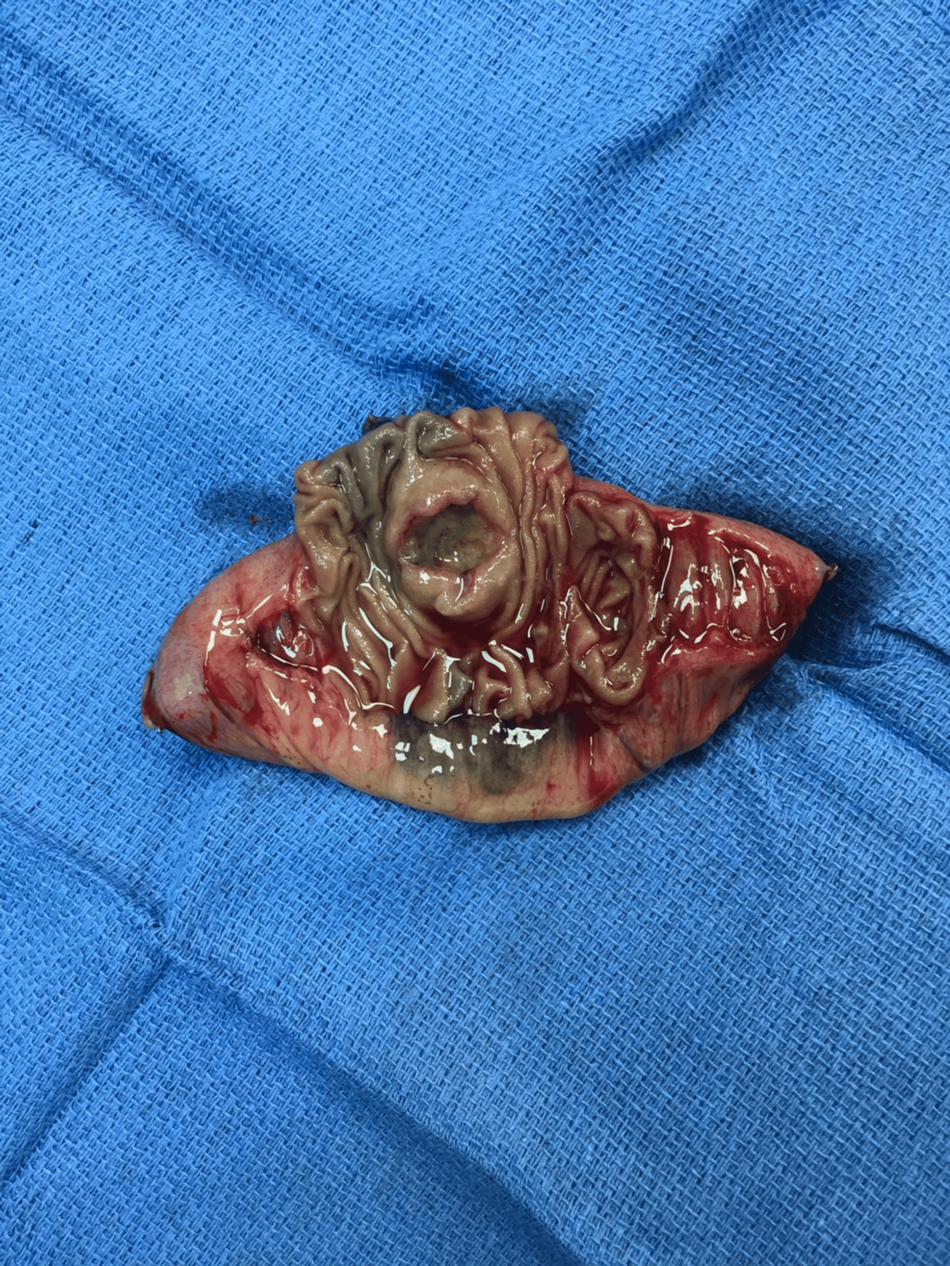
Segmental resection of the jejunum containing the lesion.

The patient underwent a laparoscopic exploration and a segmental resection of a 10 cm proximal segment of jejunum containing the lesion with primary anastomosis (side-to-side, stapled) (Figures 3, 4). The biopsy specimen revealed metastatic melanoma, infiltrating the intestinal wall. Surgical margins were free. The patient was discharged home in good clinical condition, and his hemoglobin levels increased to normal levels.

## Discussion

Metastatic melanoma is the most common primary tumor responsible for small bowel metastases, accounting for 50%-70% of such cases [11]. Despite this, hemorrhagic small bowel melanoma metastases are a rare cause of GI bleeding. Small bowel involvement is frequently missed during life, with postmortem studies revealing small bowel metastases in 50%-60% of melanoma patients, but only about 10% are diagnosed before death [12]. The terminal ileum is the most common site of small bowel melanoma metastasis, followed by the stomach.

Metastatic melanoma to the small bowel typically presents clinically with abdominal pain, anemia, overt or occult GI bleeding, and, less commonly, small bowel obstruction or perforation. In patients with a history of melanoma, new GI symptoms, especially unexplained anemia or GI bleeding, should raise suspicion for small bowel involvement. Moreover, our findings underscore the necessity for ongoing vigilance in the follow-up care of melanoma survivors. The interval between the initial diagnosis of cutaneous melanoma and the development of GI metastasis can span several years, emphasizing the need for continuous monitoring and assessment of any new GI symptoms. Healthcare providers should be equipped with the knowledge and tools to recognize the potential for metastatic spread to the GI tract and to implement appropriate diagnostic strategies, including advanced imaging techniques and endoscopic evaluations.

The recommended diagnostic approach begins with cross-sectional imaging and endoscopic evaluation. Conventional CT and routine barium studies have limited sensitivity for detecting small bowel lesions, missing up to one-third of masses, and performing especially poorly for polypoid or multiple lesions. CT may identify mural-based masses or complications, but often fails to detect smaller or flat lesions [13]. Video capsule endoscopy and enteroscopy provide direct visualization of the small bowel mucosa and are superior for localizing and characterizing intraluminal lesions, especially in cases of obscure GI bleeding or when imaging is inconclusive. Capsule endoscopy can detect bleeding sources missed by PET/CT, while deep enteroscopy allows for biopsy and therapeutic intervention. Enteroscopy has the highest diagnostic yield among modalities for suspected small bowel bleeding [14].

Surgical resection is the primary treatment modality for metastatic melanoma to the small bowel, particularly in patients with limited (oligometastatic) disease, as it is associated with increased overall survival and improved symptom control. Complete (R0) resection, when feasible, confers the greatest survival benefit. Surgery is also indicated for palliation of life-threatening complications such as bleeding, obstruction, or perforation [15].

Our case illustrates a rare presentation of overt GI bleeding due to small bowel metastasis from melanoma. The patient’s anemia led to recurrent syncope and trauma, ultimately necessitating surgical resection, which was both diagnostic and therapeutic.

## Conclusions

GI bleeding from metastatic melanoma is an uncommon yet critical clinical presentation that should be considered in patients with a prior history of melanoma. This case highlights the importance of maintaining a high index of suspicion for small bowel metastasis, especially in individuals who exhibit unexplained anemia or GI symptoms, such as melena or abdominal pain. The complexities of diagnosing and managing metastatic melanoma necessitate a multidisciplinary approach that includes oncologists, surgeons, radiologists, and pathologists. Early identification and intervention are crucial, as timely surgical resection can significantly improve patient outcomes by alleviating symptoms and potentially enhancing survival rates. In this case, the patient’s surgical intervention not only provided immediate relief from GI bleeding but also allowed for the definitive diagnosis of metastatic disease, which is pivotal for guiding further treatment options. This case serves as a reminder of the unpredictable nature of melanoma metastasis and the importance of integrating surgical interventions into the management plan for patients with a history of melanoma. Further research is warranted to explore the optimal management strategies for such presentations, which could enhance the quality of care and outcomes for patients facing similar challenges.
